# Analysis of the Status and Influencing Factors of Depression in Chinese Middle-Aged and Older Cancer Patients—Based on Empirical Evidence from the 2020 CHARLS Database

**DOI:** 10.3390/healthcare13091036

**Published:** 2025-04-30

**Authors:** Yantong Zhou, Ying Bian

**Affiliations:** 1 Institute of Chinese Medical Sciences, University of Macau, Macau, China; zhoutt2001136@163.com; 2 State Key Laboratory of Quality Research in Chinese Medicine, University of Macau, Macau, China; 3 Department of Public Health and Medicinal Administration, Faculty of Health Sciences, University of Macau, Macau, China

**Keywords:** middle-aged and older adults, cancer, depression, influencing factors, CHARLS

## Abstract

**Background/Objectives**: With the gradual acceleration of population aging in China, the health problems among middle-aged and older adults have become a key social topic. Among this segment of the population, symptoms of depression are common, and cancer has the potential to aggravate such psychological diseases. Under this background, the present study investigates depression in middle-aged and older cancer patients and the various factors that influence depression. **Methods**: Data from 356 participants (aged ≥ 45, cancer-diagnosed, CES-D score ≥ 10) were extracted from CHARLS 2020. Depressive symptoms were assessed using CES-D, with SPSS 29.0 employed for ANOVA and binary logistic regression to identify associations between cancer, covariates, and depression. **Results**: The number of depressive symptoms was 161 (45.2%). Binary logistic regression showed that “gender”, “retired status”, “social status”, “life satisfaction”, “self-rated health”, and “sleep duration” were the main factors. “Female”, “not retired”, “unsocialized”, “satisfied”, “very poor self-rated health”, and “sleep < 6 h” were found to exacerbate depressive symptoms in middle-aged and older cancer patients. **Conclusions**: Depressive symptoms are more severe in middle-aged and older cancer patients than in other groups and are influenced by various factors. Consequently, greater attention should be paid to the mental health of these patients in their daily lives, and targeted measures should be taken to improve their mental health considering all aspects of family and society. These measures may alleviate the psychological harm that these patients suffer in the process of cancer and its treatment.

## 1. Introduction

The global population is undergoing an aging process, with a concomitant increase in the number and proportion of older adults in almost every country worldwide. The 2022 World Population Prospects demonstrate that the population aged 65 and over is growing at a faster rate than the population aged under 65 and is projected to increase from 10% of the global population in 2022 to 16% in 2050. By that time, the global population aged 65 and over will have doubled compared to the population of children under 5 and will almost reach the same size as the population of children under 12 [1]. China is home to the largest older population globally, and as the “second baby boom generation” begins to retire in 2022, the country is set to witness a further increase in its aging burden. China’s “second baby boom generation” began in 1962, after the end of the three-year natural disaster. This wave peaked in 1965 and lasted until 1973, making it the most important baby boom in the country’s history, with the largest number of births and the greatest impact on the economy. By the end of 2023, China’s older population aged 65 and over was projected to account for 216.76 million individuals, representing 15.4% of the total population, thereby reaching the United Nations’ “moderate ageing” level [2]. Furthermore, the National Cancer Center of China estimated that the crude incidence rate of all cancers in China would be 341.75 per 100,000 people in 2022, representing an increase of 256,000 cancer cases compared to previous estimates—a figure largely attributable to the aging population [3]. Additionally, a study conducted by Chinese scientists identified cancer as the leading cause of hospitalization among older inpatients [4].

As the global population continues to age, the mental health of older adults must be afforded greater attention. In 2012, depression became the second most prevalent disease burden in China. According to projections by the World Health Organization, this ailment is expected to present a major global disease burden by 2030 [5]. A recent survey revealed that the prevalence of depression ranges from 1.5% to 7.9%, with the prevalence of severe depressive symptoms ranging from 1.5% to 60.3%. Additionally, depression in older adults was found to exert a substantial negative impact on Chinese society, as evidenced by the presence of comorbidity with physical illness, functional disability, and suicide risk [6,7,8]. Research has identified gender, residential status, marital status, and socioeconomic status as factors influencing the risk of depression among older adults [9]. Additionally, chronic diseases are recognized as a significant cause of depression in old age, with particular risks associated with diabetes, kidney disease, stroke, and chronic obstructive pulmonary disease [10,11,12,13]. Furthermore, cancer was shown to have a substantial impact on the mental health of older individuals. It is, therefore, imperative to acknowledge the potential for patients to encounter emotional distress during cancer treatment. This phenomenon is attributable to the adverse effects of treatment on quality of life, body image, and financial status, which have the capacity to exacerbate pre-existing mental health conditions [14,15]. Consequently, it is crucial to prioritize the mental well-being of older adults afflicted with cancer.

Recent studies have examined the association between depression and activities of daily living, cognitive ability, and specific diseases among older adults [16,17,18]. However, there is a paucity of research on the mental health of older cancer patients. For instance, there is little research on the factors affecting depression in cancer patients generally or how to protect and maintain the mental health of middle-aged and elderly cancer patients. This study utilized the China Health and Retirement Longitudinal Study (CHARLS) 2020 to comprehensively assess the mental health status of middle-aged and older cancer patients in China, with a specific focus on identifying the influential factors associated with depression. The present investigation involved an analysis of the mental health status of cancer patients in the target demographic, complemented by a comparative analysis of depression rates among elderly individuals without cancer. This approach was adopted to elucidate the impact of cancer on depression in older adults. Notably, all participants involved in this study provided written informed consent. The objective of this study is to comprehensively assess the depression status of middle-aged and older Chinese cancer patients and identify the factors contributing to their depression, with a view to exploring effective ways to alleviate depressive symptoms.

## 2. Materials and Methods

### 2.1. Population

**Figure 1:**
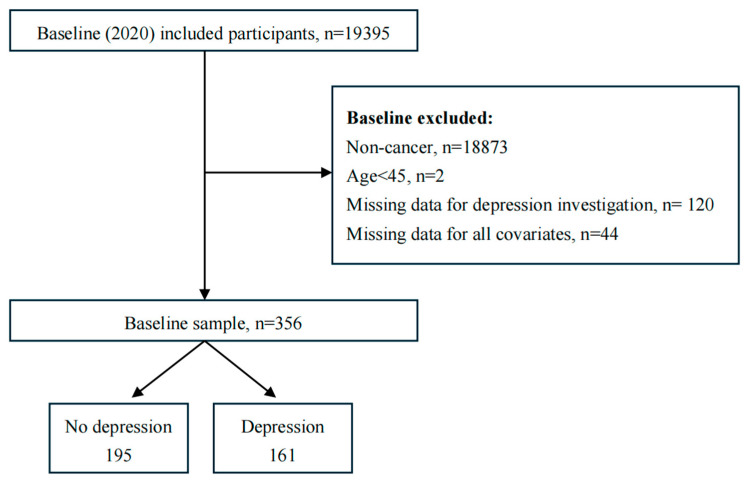
Flowchart of study participants.

The sample for this survey was drawn from the fifth round of national surveys conducted in 2020 by the China Health and Retirement Longitudinal Study (CHARLS), a tracking survey representative of the mainland Chinese population aged 45 years and older. The overarching aim of CHARLS is to build a high-quality public micro-database collecting multidimensional information covering socioeconomic status and health status to meet the needs of scientific research on aging. To ensure the representativeness of the sample, the CHARLS baseline survey used multi-stage (county/district–village/residence–household) PPS random sampling based on implicit stratification (the stratification indicators are region, urban/rural attributes, and GDP per capita), which covered 150 countries/regions and 450 villages/urban communities nationwide, involving 17,705 people in 10,257 households, thereby reflecting the overall situation of the middle-aged and older population in China [19]. Furthermore, CHARLS is a tracking survey; as the study continues, the age of the respondents will increase if new samples are not added, which will result in the younger cohorts no longer being represented. Accordingly, the aggregate number of respondents (primary respondents in addition to their spouses) increased from 17,708 in the baseline survey to 19,395 in Wave 5. The criteria for selecting respondents for this study were as follows: Firstly, respondents were required to be above the age of 45. Secondly, respondents were required to have data from the Center for Epidemiologic Studies Depression Scale (CES-D) provided by CHARLS. Thirdly, respondents must have selected “Yes” in the “Cancer” column. Finally, independent variables were selected based on valid responses to questions regarding gender, age, place of residence, and marital status. A detailed flowchart of the sample selection process is shown in Figure 1.

The total number of respondents for this survey was determined to be 356. Basic information on the participants is presented in Table 1.

CHARLS received approval from the Peking University Biomedical Ethics Committee for this round of surveys, with the fieldwork programmed for this round of household questionnaires approved under approval number: IRB00001052-11015. Informed consent was obtained from all respondents.

### 2.2. Assessment of Depressive Symptoms

Depression was measured using the CES-D rating scale, which was previously used to assess depressive symptoms in the Chinese population [17]. The CES-D questionnaire consists of 10 items that assess the respondent’s feelings and behaviors during the past week; these items were administered and completed by the CHARLS investigator. Each item has four options: (1) little or no time (<1 day), scored 0; (2) some or a small amount of time (1–2 days), scored 1; (3) occasional or a moderate amount of time (3–4 days), scored 2; and (4) most or all the time (5–7 days), scored 3. The overall score on the CES-D questionnaire is 30, and a cutoff score ≥ 10 was used to identify respondents with depressive symptoms, following routine practice in the existing literature [20]. The CES-D questionnaire presented good internal consistency, with a Cronbach alpha of 0.78. Additionally, the CES-D questionnaire was previously validated in a study on the Chinese elderly population, demonstrating its satisfactory reliability and validity [21,22].

### 2.3. Other Variables

Gender, age, current address, marital status, education level, current alcohol use, current smoking, retired status, health insurance, pension insurance, social activities, life satisfaction, self-assessment of health, sleep duration, and number of chronic diseases were selected as covariates. The definition of “social activities” was based on the question “Have you engaged in any of the following social activities in the past month?” in Section 4 of the questionnaire, Health Status and Functioning DA038. Participation in any of these activities was counted as socialization. The definition of “sleep duration” was based on the question “Have you engaged in any of the following social activities in the past month?” “Sleep duration” was defined based on each participant’s hours of actual sleep at night (the average number of hours in a night) during the past month, categorized in this study as <6 h, 6–8 h, and ≥8 h.

### 2.4. Statistical Methods

Statistical analysis of the data was conducted utilizing SPSS29.0 software (IBM Corporation). Initially, univariate analysis was employed to compare the differences between various indicators of depression in older adults with and without cancer. Subsequently, binary logistic regression analysis was implemented to examine the factors influencing depressive symptoms in older cancer patients. The findings were considered to be statistically significant at *p* < 0.05.

## 3. Results

### 3.1. Univariate Analysis

In total, 356 cancer patients were included in the chi-squared test. Among cancer patients, the number of depressed individuals totaled 161 (45.2%), with the following distribution of characteristics: female (67.1%), 60–74 years old (53.4%), rural (58.4%), married (83.9%), education below elementary school (47.8%), not drinking alcohol now (77.6%), not smoking now (87.6%), not retired now (82.6%), with medical insurance (95%), with pension insurance (85.7%), no social activities (57.8%), satisfied with life (80.1%), poor self-rated health (40.4%), sleep <6 h (32.8%), and a number of chronic illnesses totaling ≥ 4 (75.2%). These characteristics were the main subjects of analysis, with gender, age, education level, current address, social activities, life satisfaction, self-related health, and sleep duration presenting significantly different results (*p* ≤ 0.05). For patients without cancer, data on 15,330 participants were included in the comparative survey. Here, the number of depressed individuals was 5743 (37.5%), and all of the independent variables were significantly different, except for the variable measuring the presence or absence of a pension (Table 2).

### 3.2. Binary Logistic Regression Analysis

Binary logistic regression analyses were used to ascertain the associations between depression status (depression = 1, no depression = 0) and demographic characteristics in the present cohort of Chinese middle-aged and older cancer patients. Model 1 included all independent variables, while Model 2 included statistically significant independent variables identified in the univariate analyses above. The results of the study are presented in Table 3.

The findings of the binary logistic regression analysis indicated that gender, retirement status, social activities, life satisfaction, and self-rated health were the primary factors influencing depression. The male cohort (OR = 0.460, 95% CI = 0.276–0.766) exhibited a 0.46 times increased probability of depression compared with the female cohort; the retired group (OR = 0.333, 95% CI = 0.186–0.596) demonstrated a 0.333 times increased probability of depression compared with the non-retired group; and the sociable group (OR = 0.578, 98% CI = 0.350–0.955) exhibited a 0.578 times increased probability of depression compared with the unsociable group. Those who expressed satisfaction with their lives (OR = 3.736, 95% CI = 2.122–6.576) demonstrated a 3.736 times increased probability of depression compared with those who reported dissatisfaction. Furthermore, self-rated health (mediocre: OR = 0.404, 95% CI = 0.191–0.856; general: OR = 0.176, 95% CI = 0.083–0.371; rather or relatively good: OR = 0.094, 95% CI = 0.020–0.432; excellent: OR = 0.013, 95% CI = 0.042–0.686) was negatively associated with a risk of depression. Additionally, those with a sleep duration of 6–8 h (OR = 0.356, 95% CI = 0.206–0.617) were found to be 0.356 times more likely to be depressed than those who slept <6 h. Additionally, individuals who slept ≥ 8 h (OR = 0.415, 95% CI = 0.200–0.863) were 0.415 times more likely to be depressed than those who slept <6 h. Ultimately, the following characteristics were found to exacerbate depressive symptoms in middle-aged and older cancer patients: “female”, “not retired”, “unsocialized”, “satisfied”, “very poor self-rated health”, and “sleep duration < 6 h”.

## 4. Discussion

This study summarized the status of depressive symptoms and related factors in middle-aged and older cancer patients using the CHARLS database. The results demonstrated that 45.2% of middle-aged and older cancer patients exhibited depressive symptoms, indicating that the disease population is characterized by a higher prevalence of severe psychological disorders. This study revealed significant disparities in depressive symptoms between male and female cancer patients, with the prevalence being higher among women. This finding is consistent with the results of a study on anxiety and depression following a cancer diagnosis, which showed that female cancer patients were twice as likely to experience clinically significant anxiety and depression than male cancer patients [23]. Furthermore, a study investigating the impact of tumor stage on anxiety and depression in men and women revealed that depression scores for women increased more substantially with advancing tumor stage than those for men [24]. This discrepancy may be attributable to the influence of religion and spirituality on the sexes. Compared with male cancer patients, female cancer patients were found to exhibit increased spiritual enrichment and engage in more profound reflection on their lives and families. A study conducted in the United States administered the Coping Effectiveness Scale and the religious coping subscale from the Fetzert/National Institute on Aging Brief Multidimensional Measure of Religiousness/Spirituality to participants of different genders. Despite the absence of any discernible disparities in the efficacy of coping mechanisms employed, female subjects demonstrated a greater propensity to utilize religious coping strategies compared with their male counterparts [25]. Furthermore, females were observed to demonstrate more positive coping responses to cancer treatment, express their emotions through venting, and seek social support more frequently [26]. A further study examining rectal cancer revealed that female patients more frequently adopted a “fighting spirit” in their approach to dealing with the disease [27]. Conversely, men more commonly adopted a “despair/helplessness” or “compromise/acceptance” style of coping. Consequently, healthcare professionals should prioritize the mental health of middle-aged and adult female cancer patients.

The present findings indicate that retirement status exerts an influence on depressive symptoms in middle-aged and older adults diagnosed with cancer, with retired middle-aged and older adults exhibiting a reduced risk of depression compared with their non-retired counterparts. A number of studies have indicated that stressful work environments in midlife may contribute to the exacerbation of depression in later life [28]. However, empirical studies have revealed two critical findings: (1) the prevalence of depression among middle-aged and older retirees reaches 28%, and (2) the transition to retirement exacerbates multifactorial risks for late-life depression. This phenomenon may be attributable to the progressive erosion of three key psychosocial resources during retirement adaptation: social role engagement, relational networks, and financial security [29,30]. These results differ from those of the present study, possibly due to the different cultures of retirement in different countries, as well as different levels of physical fitness. A study on Chinese workers revealed higher depression rates among middle-aged and older populations, where age-related physical decline reduces workplace adaptability while compounding mental health neglect [31]. Cancer diagnosis further exacerbates these occupational and life challenges, underscoring the critical need to prioritize mental health support for this demographic during their working and post-retirement years.

The results of the present study suggest that participation in social activities is associated with depressive symptoms and that middle-aged and older cancer patients who do not participate in social activities have a higher risk of developing depressive symptoms. There is growing concern that social isolation and loneliness are detrimental to health and increase the risk of various diseases [32]. A study conducted during the period of the new coronavirus pneumonia outbreak demonstrated that limited social interaction was a risk factor for depression for cancer patients, which aligns with the present study’s conclusion that the presence of both healthcare professionals and the patient’s family and friends is crucial for cancer recovery [33]. Furthermore, the consequences of cancer and its treatments result in health limitations affecting middle-aged and older adults which, in turn, limit the activities and physical functioning of these patients. The decline in physical functioning that occurs with age further reduces the range of activities for this demographic. A study examining mental health risks among older adults found that sedentary behavior was associated with high levels of loneliness [34]. Consequently, increasing social interaction and promoting physical activity among middle-aged and older cancer patients may serve as a potential intervention strategy to alleviate depressive symptoms [35].

This study posits that cancer patients in the middle and older age groups with elevated levels of life satisfaction (LS) are more prone to depression. LS is a pivotal indicator for quality of life among older adults. However, a contradictory result was observed, suggesting that a decline in well-being among older Chinese hypertensive patients significantly impacts their LS. Consequently, enhancing well-being and quality of life in this demographic may reduce depressive symptoms [36]. The potential for conflicting conclusions may be attributable to variations in the types of diseases investigated. Firstly, the difference in causality among the studies is a contributing factor. This study utilized LS as an independent variable, whereas the previous study employed LS as a dependent variable. Additionally, this study focused on cancer, thereby limiting the results to middle-aged and older cancer patients. Consequently, a subsequent study could adopt LS as a dependent variable to elucidate the association between depression and LS. A previous study explored the mediating role of electronic devices and LS in depression among older adults and found that increasing one’s ability to use electronic devices to counteract limitations in daily functioning due to aging can increase LS and thus reduce depression [37]. In addition, a longitudinal study showed a significant increase in LS over time and a reduction in depressive symptoms among older adults [38], which is consistent with the results of the present study. Consequently, further exploration is warranted to ascertain the causal relationship between LS and depressive symptoms in middle-aged and older adults diagnosed with cancer.

The findings indicate that middle-aged and older cancer patients have a heightened risk of depression when their self-rated health is perceived to be suboptimal. Self-rated health (SRH) is defined as an individual’s subjective assessment of their own health status and has been extensively endorsed by the World Health Organization for use by individuals to assess their health [39]. Among middle-aged and older adults diagnosed with cancer, physical functioning declines to varying degrees as the disease progresses and treatment advances. Therefore, perceptions of SRH also decline among middle-aged and older adults with cancer. A study of symptoms of depression and stress and their association with oral health status showed an association between poor SRH and psychosocial symptoms in cancer patients. Indeed, poor SRH has been identified as a risk factor for anxiety and stress comorbidities in cancer patients. However, this association was not observed with depression [40]. Other studies have examined the relationship between SRH and depression, focusing on cancer survivors. For instance, depressive affect was found to be significantly associated with lower SRH among US cancer survivors [41]. Consequently, in addition to the focus on SRH outcomes for cancer survivors, greater emphasis could be placed on the association between SRH and depression in middle-aged and older cancer patients.

Sleep duration (the average number of hours slept per night) also affects depressive symptoms in middle-aged and older cancer patients. The present results showed that the risk of depression was highest for respondents who slept <6 h and lowest for those who slept 6–8 h. Those who slept ≥8 h were also at a higher risk of depression than those who slept 6–8 h. These findings align with those of other studies demonstrating an association between sleep duration and both physical health and cancer incidence, with both extended and reduced sleep durations having the potential to impact health [42]. Furthermore, it was demonstrated that both insufficient and excessive sleep can result in diminished cognitive function, thereby compromising one’s ability to effectively process emotional signals. This, in turn, can engender an increased propensity towards negative emotions during the process of emotional recognition, emotional experience, and daily communication. Moreover, such conditions are often accompanied by the development of depressive symptoms [43,44,45]. A study examining the impact of sleep duration on depression in older adults further substantiated these findings, demonstrating that a short sleep duration (<6 h) is associated with increased depression scores and that a sleep duration of 6–8 h may not represent the optimal sleep duration for older adults [46]. The present study concluded that maintaining a moderate sleep duration is beneficial for reducing depressive symptoms in middle-aged and older cancer patients. However, there is a paucity of research on the relationship between depressive symptoms and specific sleep durations among middle-aged and older cancer patients, as most studies focus on the relationship between sleep duration and depression.

The innovation of this study lies in its focus on cancer patients as the primary subjects, utilizing the CHARLS database to investigate the depressive symptoms of middle-aged and older adults in China, along with the factors that influence these symptoms. This aspect has not been previously addressed in the literature. In terms of data analysis, the present study compared the associations between middle-aged and older cancer patients, as well as patients without cancer, and depression. For patients without cancer, significant covariates were higher in number, possibly because the sample size of middle-aged and older adults without cancer was larger than that of cancer patients, resulting in a more significant difference.

However, the present study also has some limitations. The investigation was confined to cross-sectional data from CHARLS 2020 pertaining to that particular year, and thus we cannot rule out the possibility that data are missing due to the nature of the CHARLS database as a tracking survey conducted every two to three years. Additionally, the survey’s focus on women, rural populations, and individuals with limited education levels may have introduced bias into the results. Consequently, in subsequent studies, the Depression Survey of middle-aged and older cancer patients could be applied longitudinally, and the factors affecting the positive loading of depression could be explored in more detail by combining data from the previous CHARLS.

## 5. Conclusions

The present survey revealed that 45.2% of Chinese middle-aged and older cancer patients exhibited symptoms consistent with depression. The prevalence of depression among this demographic was found to be associated with gender, retirement status, social activities, life satisfaction, self-assessed health, and sleep time. Consequently, to enhance the quality of life and mental well-being of middle-aged and older cancer patients in China, it is imperative to allocate greater attention to the activities and life circumstances of middle-aged and older individuals following their retirement. Moreover, it is crucial to promote the active involvement of healthcare professionals, community representatives, and family members in advocating for the mental health and well-being of middle-aged and older individuals. This measure would increase time spent in the company of older individuals, enabling them to feel more socially engaged. Additionally, it is crucial to monitor the sleep patterns of middle-aged and older cancer patients, in order to better maintain their psychological well-being and reduce the risk of depression during their cancer treatment period. Finally, it is imperative to regulate the sleep duration of middle-aged and older cancer patients to promote a healthy psychological state and mitigate emotional distress during the cancer treatment period. This approach could also indirectly reduce the impact of depression on the disease.

## Figures and Tables

**Table 1:** Basic information on the study subjects.

Variable	Number (N)	Percentage (%)
**Gender**		
Male	148	41.6
Female	208	58.4
**Age**		
45–59	128	36.0
60–74	188	52.8
75 and over	40	11.2
**Current address**		
Urban	166	46.6
Rural	190	53.4
**Marital status**		
Married	309	86.8
Else	47	13.2
**Educational level**		
Below elementary school	133	37.4
Secondary schools	91	25.6
Middle school	71	19.9
High school and above	61	17.1
**Current alcohol use**		
Yes	83	23.3
No	273	76.7
**Current smoking**		
Yes	49	13.8
No	307	86.2
**Retired status**		
Retired	100	28.1
Not retired	256	71.9
**Medical insurance**		
Yes	345	96.9
No	11	3.1
**Pension insurance**		
Yes	308	86.5
No	48	13.5
**Social activities**		
Sociable	171	48.0
Unsocialized	185	52.0
**Life satisfaction**		
Satisfied	238	66.9
Dissatisfied	118	33.1
**Self-related health**		
Poor	61	17.1
Mediocre	121	34.0
General	138	38.8
Rather or relatively good	19	5.3
Excellent	17	4.8
**Sleep duration**		
<6 h	157	44.1
6–8 h	142	39.9
≥8 h	57	16.0
**Total number of chronic diseases**		
1	25	7.0
2	30	8.4
3	45	12.6
≥4	256	71.6

**Table 2:** Univariate analysis of depression status in middle-aged and older adults with and without cancer.

Variable	Cancer				No Cancer			
	No Depression (195)	Depression (161)	X^2^	*p*-Value	No Depression (9587)	Depression (5743)	X^2^	*p*-Value
Gender			9.062	0.003 **			441.966	<0.001 ***
Male	95 (48.7%)	53 (32.9%)			5263 (54.9%)	2146 (37.4%)		
Female	100 (51.3%)	108 (67.1%)			4324 (45.1%)	3597 (62.6%)		
Age			6.272	0.043 *			91.302	<0.001 ***
45–59	64 (32.8%)	64 (39.8%)			4453 (46.4%)	2222 (38.7%)		
60–74	102 (52.3%)	86 (53.4%)			4210 (43.9%)	2833 (49.3%)		
75 and over	29 (14.9%)	11 (6.8%)			924 (9.6%)	688 (12.0%)		
Current address			2.970	0.085			280.548	<0.001 ***
Urban	99 (50.8%)	67 (41.6%)			4324 (45.1%)	1804 (31.4%)		
Rural	96 (49.2%)	94 (58.4%)			5263 (54.9%)	3939 (68.6%)		
Marital status			2.227	0.136			159.776	<0.001 ***
Married	174 (89.2%)	135 (83.9%)			8525 (88.9%)	4689 (81.6%)		
Else	21 (10.8%)	26 (16.1%)			1062 (11.1%)	1054 (18.4%)		
Educational level			15.839	0.001 **			612.015	< 0.001 ***
Below elementary school	56 (28.7%)	77 (47.8%)			3148 (32.8%)	2888 (50.3%)		
Secondary schools	57 (29.2%)	34 (21.1%)			2183 (22.8%)	1327 (23.1%)		
Middle school	40 (20.5%)	31 (19.3%)			2613 (27.3%)	1076 (18.7%)		
High school and above	42 (21.5%)	19 (11.8%)			1643 (17.1%)	452 (7.9%)		
Current alcohol use			0.150	0.699			252.686	<0.001 ***
Yes	47 (24.1%)	36 (22.4%)			4102 (42.8%)	1718 (29.9%)		
No	148 (75.9%)	125 (77.6%)			5485 (57.2%)	4025 (70.1%)		
Current smoking			0.446	0.504			91.776	<0.001 ***
Yes	29 (14.9%)	20 (12.4%)			2813 (29.3%)	1279 (22.3%)		
No	166 (85.1%)	141 (87.6%)			6774 (60.3%)	4464 (77.7%)		
Retired status			16.655	<0.001 ***			259.575	<0.001 ***
Retired	72 (36.9%)	28 (17.4%)			2080 (21.7%)	655 (11.4%)		
Not retired	123 (63.1%)	133 (82.6%)			7506 (78.3%)	5088 (88.6%)		
Medical insurance			3.466	0.063			9.340	0.002 **
Yes	192 (98.5%)	153 (95%)			9221 (96.2%)	5465 (95.2%)		
No	3 (1.5%)	8 (5%)			366 (3.8%)	278 (4.8%)		
Pension insurance			0.162	0.687			1.385	0.239
Yes	170 (87.2%)	138 (85.7%)			8257 (86.1%)	4907 (85.4%)		
No	25 (12.8%)	23 (14.3%)			1330 (13.9%)	836 (14.6%)		
Social activities			3.958	0.047 *			37.955	<0.001 ***
Sociable	103 (52.8%)	68 (42.2%)			5025 (52.4%)	3028 (52.7%)		
Unsocialized	92 (47.2%)	93 (57.8%)			4562 (47.6%)	2715 (47.3%)		
Life satisfaction			23.358	<0.001 ***			482.583	<0.001 ***
Satisfied	109 (55.9%)	129 (80.1%)			4030 (42.0%)	1407 (24.5%)		
Dissatisfied	86 (44.1%)	32 (19.9%)			5557 (58.0%)	4336 (75.5%)		
Self-related health			43.380	<0.001 ***			1750.196	<0.001 ***
Poor	16 (8.2%)	45 (28.0%)			242 (2.5%)	712 (12.4%)		
Mediocre	56 (28.7%)	65 (40.4%)			1093 (11.4%)	1534 (26.7%)		
General	94 (48.2%)	44 (27.3%)			5008 (52.2%)	2823 (49.2%)		
Rather or relatively good	16 (8.2%)	3 (1.9%)			1623 (16.9%)	370 (6.4%)		
Excellent	13 (6.7%)	4 (2.5%)			1621 (16.9%)	304 (5.3%)		
Sleep duration			22.284	<0.001 ***			818.026	<0.001 ***
<6 h	64 (32.8%)	93 (57.8%)			2520 (32.8%)	2813 (49.0%)		
6–8 h	94 (48.2%)	48 (29.8%)			4634 (48.2%)	1973 (34.4%)		
≥8 h	37 (19.0%)	20 (12.4%)			2433 (19.0%)	957 (16.7%)		
Total number of chronic diseases			1.532	0.675			770.117	<0.001 ***
1	15 (7.7%)	10 (6.2%)			4801 (50.1%)	1796 (31.3%)		
2	18 (9.2%)	12 (7.5%)			1960 (20.4%)	1150 (20.0%)		
3	27 (13.8%)	18 (11.2%)			1316 (13.7%)	927 (16.1%)		
≥4	135 (69.2%)	121 (75.2%)			1510 (15.8%)	1870 (32.6%)		

(* *p* < 0.05, ** *p* < 0.01, *** *p* < 0.001; Bolded text indicates statistically significant results). The X^2^ value is the core statistic in the chi-squared test and used to indicate the degree of difference between the observed and expected frequencies; the *p*-value is the probability of observing the current sample data or a more extreme situation occurring when the null hypothesis is true.

**Table 3:** Binary logistic regression analysis of the influencing factors of depression in middle-aged and older adults with cancer.

Variable	Depression (No Depression = 0, Depression = 1)			
	Model 1		Model 2	
	*p*	OR (95% CI)	*p*	OR (95% CI)
**Gender** (female as a reference)				
Male	0.008	0.401 (0.204–0.792)	0.003	0.460 (0.276–0.766)
**Retired status** (not retired as a reference)				
Retired	0.006	0.351 (0.166–0.740)	<0.001	0.333 (0.186–0.596)
**Social activities** (no socialization as a reference)				
Sociable	0.027	0.508 (0.279–0.924)	0.033	0.578 (0.350–0.955)
**Life satisfaction** (dissatisfied as a reference)				
Satisfied	<0.001	4.411 (2.397–8.117)	<0.001	3.736 (2.122–6.576)
**Self-related health** (poor as a reference)				
Mediocre	0.031	0.420 (0.191–0.926)	0.018	0.404 (0.191–0.856)
General	<0.001	0.181 (0.082–0.400)	<0.001	0.176 (0.083–0.371)
Rather or relatively good	<0.001	0.058 (0.011–0.306)	0.002	0.094 (0.020–0.432)
Excellent	0.013	0.154 (0.035–0.677)	0.013	0.169 (0.042–0.686)
**Sleep duration** (<6 h as a reference)				
6–8 h	<0.001	0.382 (0.218–0.668)	<0.001	0.356 (0.206–0.617)
≥8 h	0.021	0.415 (0.197–0.877)	0.019	0.415 (0.200–0.863)
**Age** (45–59 as a reference)				
60–74 years	0.783	1.090 (0.592–2.007)	-	-
75 and over	0.251	0.555 (0.203–1.517)	-	-
**Education level** (less than elementary school as a reference)				
Secondary schools	0.018	0.440 (0.222–0.870)	-	-
Middle school	0.573	0.802 (0.371–1.730)	-	-
High school and above	0.124	0.502 (0.209–1.209)	-	-
Place of residence (urban as a reference)				
Rural	0.952	0.981 (0.523–1.841)	-	-
**Marital status** (other as a reference)				
Married	0.071	0.477 (0.213–1.065)	-	-
**Current alcohol use** (no as a reference)				
Yes	0.085	1.871 (0.917–3.819)	-	-
**Current smoking** (no as a reference)				
Yes	0.526	1.1319 (0.561–3.098)	-	-
**Health insurance** (no as a reference)				
Yes	0.108	0.257 (0.049–1.350)	-	-
**Pension insurance** (no as a reference)				
Yes	0.527	1.286 (0.590–2.805)	-	-
**Number of chronic diseases** (1 as a reference)				
2	0.417	0.584 (0.159–2.139)	-	-
3	0.510	0.659 (0.191–2.277)	-	-
≥4	0.886	0.924 (0.313–2.725)	-	-

*p* is the level of significance; OR and 95% CI indicate a multiplicative change in the odds of the dependent variable occurring for each unit change in the independent variable and its level of confidence.

## Data Availability

The datasets used and analyzed in the present study are available upon reasonable request from the official CHARLS (https://charls.pku.edu.cn/en/). (Accessed on 30 July 2024).

## Abbreviations

The following abbreviations are used in this manuscript:

CHARLS	China Health and Retirement Longitudinal Study
CES-D	Center for Epidemiologic Studies Depression Scale
LS	Life satisfaction
SRH	Self-rated health
